# Supplementary material for: Content-rich biological network constructed by mining PubMed abstracts
Source: BMC Bioinformatics. 2004 Oct 8;5:147. doi: 10.1186/1471-2105-5-147 (PMC528731; doi:10.1186/1471-2105-5-147)
Supplement: Additional File 2 — The original results of the above study (non-essential files are deleted to keep the file size under the limit set by BMC bioinformatics). [file 1471-2105-5-147-S2.bz2 › chilibotAdditionalFile2/dip10/600ID10092459E10979/html/PHES_PHET.html]

 


 **PHES** and **PHET** 
  
Found 54 abstracts in PubMed, retrieved 10.  
 

 What does Google say? 
 PDF only 
| .edu only 

---

**Interactive relationship** (e.g. stimulation, inhibition, etc)

**Neutral relationship**- In prokaryotes, the alpha and beta subunits of pheRS  [ **PHET** ]   [ **PHES** ]  are encoded by the genes  **pheS**  and  **pheT** , respectively.  Ref: 11281723 Protein Expr Purif, 2001
- Tn5luxAB was localized to a fragment of chromosomal DNA encoding the thrS, infC, rpmI, rplT,  **pheS**  and  **pheT**  genes, with Tn5 inserted in the 3 end of  **pheS** .  Ref: 12583892 FEMS Microbiol Lett, 2003
- A sulfate ion, which was identified on the protein surface, may mediate the interactions of PheRS  [ **PHET** ]   [ **PHES** ]  with DNA.  Ref: 11679717 Acta Crystallogr D Biol Crystallogr, 2001

**Non-interactive relationship** (e.g. studied together, co-existance, homology, etc.)

- The h mt TrpRS shows kinetic properties similar to human mitochondrial phenylalanyl tRNA synthetase h mt PheRS  [ **PHET** ]   [ **PHES** ] , and h mt TrpRS is not induced by interferon gamma as is hTrpRS.  Ref: 10828066 J Biol Chem, 2000
- The extent of tRNA recognition at the level of binding by Thermus thermophilus phenylalanyl tRNA synthetase PheRS  [ **PHET** ]   [ **PHES** ] , one of the most complex class II synthetases, has been studied by independent measurements of the enzyme association with wild type and mutant tRNA Phe s as well as with non cognate tRNAs.  Ref: 12382236 J Mol Recognit< MedlineTA>J Mol Recognit, 2000
- The results suggest specific binding of the 3 ; terminal nucleotide of tRNAPhe by the catalytic subunit of PheRS  [ **PHET** ]   [ **PHES** ]  in the absence of other substrates.  Ref: 11092959 Biochemistry Mosc , 2000
- Starting with the crystal structure of Thermus thermophilus PheRS  [ **PHET** ]   [ **PHES** ]  without bound ligand, HierDock predicts the binding site of phenylalanine Phe within 1.1 A of that revealed by the crystal structure of PheRS  [ **PHET** ]   [ **PHES** ]  cocrystallized with Phe.  Ref: 12452720 J Am Chem Soc, 2002
- We describe the high level overexpression and purification of recombinant S. aureus pheRS  [ **PHET** ]   [ **PHES** ]  using  **pheS**  and  **pheT**  genes as part of an artificial operon in Escherichia coli.  Ref: 11281723 Protein Expr Purif, 2001
- In this paper, we describe a computational protocol HierDock for predicting the relative energies of binding of phenylalanine analogues to phenylalanyl tRNA synthetase PheRS  [ **PHET** ]   [ **PHES** ] .  Ref: 12452720 J Am Chem Soc, 2002
- In particular, it is suggested that the monomeric PheRS  [ **PHET** ]   [ **PHES** ]  from the yeast mitochondrion is a chimera of the alpha and beta chains of the standard tetrameric protein.  Ref: 12572618 Mol Biol Evol, 1998
- We also revealed a direct interaction of PheRS  [ **PHET** ]   [ **PHES** ]  with eEF1A in the absence of tRNAPhe Kd = 21 nm.  Ref: 12354112 Eur J Biochem, 2002
- The interaction of Thermus thermophilus phenylalanyl tRNA synthetase PheRS  [ **PHET** ]   [ **PHES** ]  with the 3 ; terminal nucleotide of tRNAPhe has been studied by affinity labeling to solve the problem arising from X ray crystallographic study the binding sites of phenylalanine and the 3 ; terminal nucleotide base were revealed to be identical in the crystal structures of PheRS  [ **PHET** ]   [ **PHES** ]  complexed with the substrates.  Ref: 11092959 Biochemistry Mosc , 2000
- For comparative analysis we also report a procedure for the purification of native pheRS  [ **PHET** ]   [ **PHES** ]  from S. aureus Oxford Strain and demonstrate that Michaelis Menten parameters for both recombinant and native enzyme, at least for phenylalanine tRNA aminoacylation are comparable.  Ref: 11281723 Protein Expr Purif, 2001
- Identification, cloning, and expression of a functional phenylalanyl tRNA synthetase pheRS  [ **PHET** ]   [ **PHES** ]  from Staphylococcus aureus.  Ref: 11281723 Protein Expr Purif, 2001
- A few backbone mediated contacts of PheRS  [ **PHET** ]   [ **PHES** ]  with the acceptor and anticodon stems revealed in the crystal structure do not contribute to tRNA Phe discrimination, their role being limited to stabilization of the complex.  Ref: 12382236 J Mol Recognit< MedlineTA>J Mol Recognit, 2001
- The calculated binding energies of Phe analogues in PheRS  [ **PHET** ]   [ **PHES** ] , using HierDock, correlate well with the translational activities of the same analogues in Escherichia coli.  Ref: 12452720 J Am Chem Soc, 2002
- Aminoacylation activity of the overproduced human PheRS  [ **PHET** ]   [ **PHES** ]  in the crude bacterial extract is two orders of magnitude higher than the corresponding activity in human placenta and the yield of the recombinant enzyme overproduced in E. coli is five times higher.  Ref: 11858721 Protein Expr Purif, 2002
- Crystals of native PheRS  [ **PHET** ]   [ **PHES** ]  were soaked in a solution containing phenylalanine and ATP in the presence of Mn 2 ions.  Ref: 11679717 Acta Crystallogr D Biol Crystallogr, 2001
- The dissociation constants for the eEF1A.GDP.tRNA and eEF1A.GDP.tRNAPhe.PheRS  [ **PHET** ]   [ **PHES** ]  complexes were found to be 20 nm and 9 nm, respectively.  Ref: 12354112 Eur J Biochem, 2002
- Visible conformational changes were detected in the active site area adjacent to the position of the Phe AMP, compared with the structure of PheRS  [ **PHET** ]   [ **PHES** ]  complexed with a synthetic adenylate analogue phenylalaninyl adenylate.  Ref: 11679717 Acta Crystallogr D Biol Crystallogr, 2001
- Phenylalanyl tRNA synthetase pheRS  [ **PHET** ]   [ **PHES** ]  is unique among aminoacyl tRNA synthetases in that it is a heterotetrameric enzyme composed of two alpha subunits and two larger beta subunits.  Ref: 11281723 Protein Expr Purif, 2001
- Comparative analysis of the cross linked products in the absence and in the presence of small substrates revealed ATP and aminoacyl adenylate to effect the interaction of the tRNAPhe acceptor end with PheRS  [ **PHET** ]   [ **PHES** ] .  Ref: 11092959 Biochemistry Mosc , 2000
- HierDock identifies p fluorophenylalanine and 3 thienylalanine as especially good substrates for PheRS  [ **PHET** ]   [ **PHES** ] , in agreement with experiment.  Ref: 12452720 J Am Chem Soc, 2002
- The crystal structure of phenylalanyl tRNA synthetase PheRS  [ **PHET** ]   [ **PHES** ]  from Thermus thermophilus, a class II aminoacyl tRNA synthetase, complexed with phenylalanyl adenylate Phe AMP was determined at 2.6 A resolution.  Ref: 11679717 Acta Crystallogr D Biol Crystallogr, 2001
- Human phenylalanyl tRNA synthetase PheRS  [ **PHET** ]   [ **PHES** ]  was cloned and expressed in Escherichia coli.  Ref: 11858721 Protein Expr Purif, 2002
- The highest affinity of T. thermophilus PheRS  [ **PHET** ]   [ **PHES** ]  for cognate tRNA, observed for synthetase tRNA complexes, results in 100 3000 fold binding discrimination against non cognate tRNAs.  Ref: 12382236 J Mol Recognit< MedlineTA>J Mol Recognit, 2002
- Three different types of alphabeta 2 heterodimers of human PheRS  [ **PHET** ]   [ **PHES** ]  carrying HT at the N terminus of either of two alpha or beta subunits or simultaneously on both of them were overproduced and purified.  Ref: 11858721 Protein Expr Purif, 2002
- In this report we describe the isolation of a DNA fragment 3.52 kb containing the  **pheS**  and  **pheT**  genes from a Staphylococcus aureus WCUH29 genomic DNA library.  Ref: 11281723 Protein Expr Purif, 2001
- The correct positioning of the 3 ; terminal nucleotide of tRNAPhe corresponding to the structure of the productive complex with PheRS  [ **PHET** ]   [ **PHES** ]  is therefore promoted only in the presence of all three substrates.  Ref: 11092959 Biochemistry Mosc , 2000
- The data obtained, combined with kinetic data on aminoacylation, clearly show that PheRS  [ **PHET** ]   [ **PHES** ]  exhibits more tRNA selectivity at the level of binding than at the level of catalysis.  Ref: 12382236 J Mol Recognit< MedlineTA>J Mol Recognit, 2000
- Here we present the data on association of the eEF1A.GDP.tRNA complex with phenylalanyl tRNA synthetase PheRS  [ **PHET** ]   [ **PHES** ] , e.g. the formation of the above quaternary complex detected by the gel retardation and surface plasmon resonance techniques.  Ref: 12354112 Eur J Biochem, 2002
